# Diagnostic value of circulating tumor cells in patients with thyroid cancer: a retrospective study of 1478 patients

**DOI:** 10.1007/s12672-024-00976-4

**Published:** 2024-04-12

**Authors:** Qingxin Zeng, Haifeng Zhong, Hui Rao, Yuedong Wang

**Affiliations:** 1 Department of Thyroid Surgery, Meizhou People’s Hospital (Huangtang Hospital)/Meizhou Academy of Medical Sciences, No. 63, Huangtang Road, Meijiang District, Meizhou, 514031 China; 2 Department of Laboratory Medicine, Meizhou People’s Hospital (Huangtang Hospital)/Meizhou Academy of Medical Sciences, Meizhou, China

**Keywords:** Circulating tumor cells, Thyroid carcinoma, *BRAF* V600E, Liquid biopsy

## Abstract

**Background:**

Circulating tumor cell (CTC) detection is one form of liquid biopsy. It is a novel technique that is beginning to be applied in the field of thyroid cancer. The present study was designed to evaluate the diagnostic value of CTCs in patients with thyroid cancer.

**Methods:**

A total of 1478 patients were retrospectively analyzed and divided into malignant group (n = 747) and benign group (*n* = 731). Peripheral blood was collected, and CTCs were enriched and quantified before surgery. The baseline data of the two groups were matched by Propensity Score Matching (PSM). Receiver operating characteristic (ROC) curves were used to evaluate the diagnostic efficiency of different indicators for thyroid cancer. The malignant group before PSM was further divided into subgroups according to the *BRAF* V600E mutation and lymphatic metastasis (N stage), and the number of CTCs in different subgroups was compared.

**Results:**

After 1:1 PSM, baseline characteristics of the malignant group and benign group were matched and assigned 315 cases in each group. The number of CTCs and the TPOAb values were comparable in the two groups (*p* > 0.05). The TgAb values [1.890 (1.110 – 16.010) vs 1.645 (1.030 – 7.073) IU/mL, *p* = 0.049] were significantly higher in the malignant group than in the benign group. After PSM, ROC analyses showed that the areas under the curve (AUCs) of CTC, TgAb and ultrasound were 0.537 (sensitivity 65.6%, specificity 45.8%), 0.546 (sensitivity 40.0%, specificity 70.8%) and 0.705 (sensitivity 77.1%, specificity 63.2%), respectively. The AUCs of the combined detection of ‘CTC + ultrasound’ (combine 1) and the combined detection of ‘CTC + TgAb + ultrasound’ (combine 2) were 0.718 (sensitivity 79.3%, specificity 61.7%) and 0.724 (sensitivity 78.0%, specificity 63.3%), respectively. The AUC of ultrasound was significantly higher than CTC (*p* < 0.001). There was no statistically significant difference in AUC between combination 1 and ultrasound, and between combination 2 and ultrasound (*p* > 0.05). The number of CTCs between the N0 and N1 subgroups, and between the *BRAF* mutant and *BRAF* wild subgroups was comparable (*p* > 0.05).

**Conclusions:**

As an emerging and noninvasive testing tool, the efficacy of CTCs in diagnosing thyroid cancer is limited.

## Introduction

Thyroid tumor is a common and frequently occurring disease, with a higher incidence rate in females than in males [1]. Most benign thyroid tumors do not require surgical treatment, but malignant tumors are mainly treated with surgery. Differentiated thyroid carcinoma (DTC), which accounts for the largest proportion of thyroid cancer types, has a good prognosis as long as it is diagnosed early and operated timely [2]. Therefore, the focus of thyroid tumor treatment is to accurately identify benign and malignant tumors.

At present, the identification of benign and malignant thyroid tumors mainly relies on high-resolution ultrasound, contrast-enhanced ultrasonography, fine-needle aspiration (FNA) biopsy, radionuclide imaging, tests of thyroid functions and relevant antibodies [3]. The advantages of color ultrasound examination lie in its low cost and popularity, but to some extent, the results depend on the sonographer’s experience and subjective judgment. In addition, it also relies on high-end and sophisticated ultrasonic equipment for small cancer lesions. For contrast-enhanced ultrasonography, it mainly judges the benign and malignant of thyroid tumors by the blood flow in thyroid nodules. At present, there is also a view that the blood flow signal in thyroid nodules is less correlated with benign and malignant [4]. FNA is a preoperative examination for thyroid tumors with high accuracy, but it is an invasive test that makes patients feel painful and scared. There is a risk of hematoma in the neck or local exudation after aspiration, which affects the anatomical procedure and the following surgeries. In addition, pathologists must be highly experienced in cytopathology and need to attend professional training courses. Other tests, such as radionuclide imaging and tests of thyroid functions and relevant antibodies, have lower sensitivity and specificity. Currently, a large number of scholars are exploring new diagnostic methods for thyroid tumors.

Liquid biopsy is an emerging technique for the diagnosis and prognosis of tumors, which includes the detection of circulating free DNA (cfDNA), microRNA (miRNA), circulating tumor proteins and metabolites, and circulating tumor cells (CTCs) [5, 6]. It has been increasingly applied in the diagnosis and follow-up of cancers such as lung cancer, breast cancer and colorectal cancer. CTCs, cells shed from tumor tissue and released into peripheral blood, were reported as early as 1869 and have been promoted in the clinical work of thyroid cancer in recent years [7]. A meta-analysis indicated that CTC was a reliable marker for the diagnosis of thyroid cancer patients with recurrence and distant metastasis [8]. However, in most reports, CTC is mostly used to evaluate the prognosis, recurrence risk or radioactive iodine treatment effect of patients with thyroid carcinoma, and the sample size is small. The preoperative diagnostic value of CTCs for thyroid cancer is rarely reported.

Therefore, the present study aimed to evaluate the diagnostic value of CTCs in patients with thyroid cancer, so as to provide an objective reference for clinicians to apply CTC as a diagnostic tool before surgery.

## Methods

### Study design and patients

**Fig. 1:**
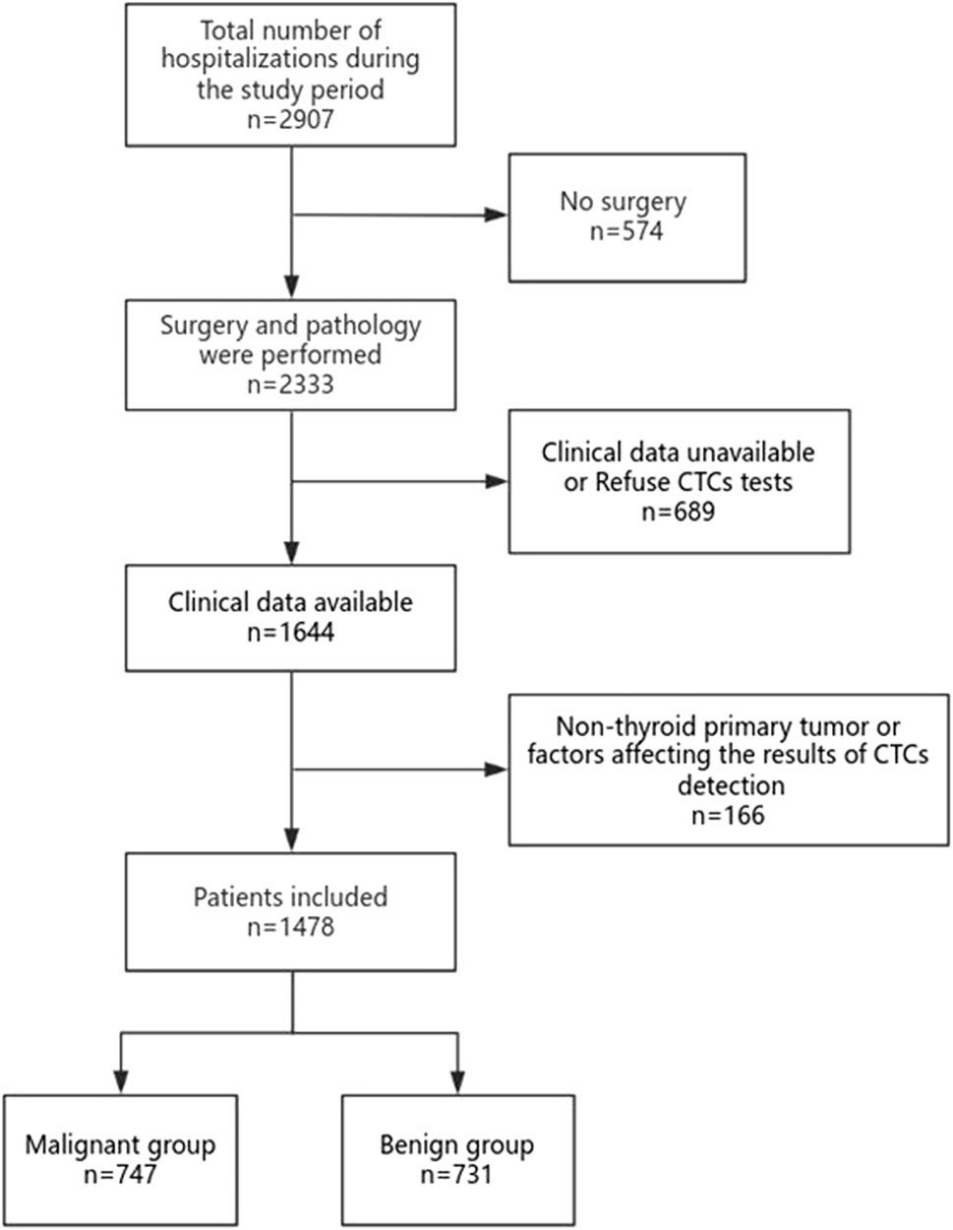
Patient flowchart

A total of 1478 patients who were hospitalized in the Department of Thyroid Surgery, Meizhou People's Hospital from January 2021 to September 2022 and met the inclusion and exclusion criteria were retrospectively analyzed (shown in Fig. 1). The inclusion criteria were as follows: (a) primary thyroid tumor; (b) clinical data including the results of preoperative thyroid color ultrasound, CTCs, thyroglobulin antibody (TgAb) and thyroid peroxidase antibody (TPOAb) were available; and (c) all patients underwent surgical treatment, and the pathological type of tumor was confirmed by postoperative pathology. The exclusion criteria were as follows: (a) obvious factors affecting the results of CTCs detection in patients, such as local or systemic infection and high or low white blood cell counts; (b) certain drugs that interfere with the detection of CTCs, such as taking large doses of folic acid; and (c) nonthyroid primary tumors, such as head and neck metastases and neurogenic tumors, etc. According to the postoperative pathological results, the patients were divided into malignant group (*n* = 747) and benign group (*n* = 731).

Thyroid color doppler ultrasound, the number of CTCs, TgAb and TPOAb were detected before surgery. The results of thyroid ultrasound were interpreted by the Chinese-TIRADS (C-TIRADS) grading system [9]. Some patients underwent FNA to assist in diagnosis.

### *Folate receptor-positive circulating tumor cell (FR* + *CTC) analysis*

CTCs were enriched and quantified using the CytoploRare Kit (Genosaber Biotech, Shanghai, China) according to the manufacturer’s instructions. In brief, 3 mL peripheral blood was first collected in EDTA anticoagulation tubes and stored at 4 ℃. CTCs were enriched by lysis of erythrocytes followed by immunomagnetic depletion of white blood cells using anti-CD45 and anti-CD14 magnetic beads. The enriched CTCs were labeled by a folate receptor (FR) α-targeting probe that contained the conjugate of a folic acid and a synthesized oligonucleotide. The labeled FR + CTCs were enumerated by quantitative polymerase chain reaction (PCR) on an ABI 7500 Real-Time PCR system (Thermo Fisher Scientific, USA). A series of standards containing oligonucleotides (10^–14^ to 10^–9^ M, corresponding to 2 to 2 × 10^5^ FU/3 mL blood) were used for FR + CTC quantification. The FR unit (FU) per 3 mL of peripheral blood was calculated from the standard curve and used to determine the FR + CTC level in each sample.

### BRAF V600E mutation detection

Paraffin-embedded sections of thyroid tumors removed after surgery were taken for digestion, histone removal, and RNA removal. DNA was extracted using a nucleic acid extraction column (Amoy Diagnostics Co., Ltd., Xiamen, China). The *BRAF* V600E mutation was detected using the Human *BRAF* V600E Mutation Assay Kit (Amoy Diagnostics Co., Ltd., Xiamen, China) and amplified by the SLAN-96S Real-Time PCR system (Hongshi, Shanghai, China). The internal control HEX (or VIC) signal of the sample to be tested should have a clear amplification curve, and the Ct value should be 13 – 21. If the FAM signal had a significant amplification curve and the Ct value was less than 28, the sample did not require repeated testing, and the result was *BRAF* V600E mutation present. If the Ct value was greater than 28, the result was *BRAF* V600E mutation absent.

### Statistical analysis

IBM SPSS 26.0 statistical software was used for data processing. The normality test of continuous variables did not conform to the normal distribution, so the Mann‒Whitney U test was used to compare the mean of the independent samples in groups. The results were expressed as the median and interquartile range [M (P25 – P75)]. Categorical data of the two groups were compared by χ^2^ test, and the results were expressed as percentage (%). Propensity Score Matching (PSM) was used for balancing the baseline of the two groups by 1:1 matching and caliper 0.02. Receiver operating characteristic (ROC) curve analysis was performed to assess the sensitivity and specificity of different diagnostic methods. Multivariate logistic analysis was adopted to evaluate the risk factors for thyroid cancer and lymph node metastasis. *p* < 0.05 was considered statistically significant.

## Results

### Clinical characteristics of patients

As shown in Table 1, whether it is thyroid cancer or benign thyroid tumor, the incidence in women is much higher than that in men. However, there was no significant difference in the gender ratio between the two groups. Before PSM, the age was significantly younger, and the tumor size was significantly smaller in the malignant group than those in the benign group at the time of receiving surgery (*p* < 0.001). Nevertheless, the number of CTCs and TgAb values in the malignant group were significantly higher than those in the benign group (*p* = 0.001, *p* < 0.001, respectively). The TPOAb values were comparable in the two groups. After 1:1 PSM, patients in the two groups were matched in age and tumor size, and assigned 315 cases in each group. The number of CTCs and the TPOAb values were comparable in the two groups after PSM. The TgAb values in the malignant group was still significantly higher than the benign group (*p* = 0.049).

**Table 1:** Clinical characteristics of patients with thyroid tumor before and after PSM

Characteristics	All	Before PSM			After PSM		
		Malignant group	Benign group	*p* value	Malignant group	Benign group	*p* value
*n*	1478	747	731	–	315	315	–
Gender				0.941			0.550
Male	290 (19.6%)	146 (19.5%)	144 (19.7%)		66 (21.0%)	60 (19.0%)	
Female	1188 (80.4%)	601 (80.5%)	587 (80.3%)		249 (79.0%)	255 (81.0%)	
Age (years)				0.000			0.362
Median	50 (40 ~ 57)	47 (36 ~ 53)	53 (44 ~ 60)		50 (38 ~ 58)	49 (39 ~ 57)	
< 45	513 (34.7%)	330 (44.2%)	183 (25.0%)		109 (34.6%)	119 (37.8%)	
45–55	513 (34.7%)	266 (35.6%)	247 (33.8%)		108 (34.3%)	105 (33.3%)	
> 55	452 (30.6%)	151 (20.2%)	301 (41.2%)		98 (31.1%)	91 (28.9%)	
Tumor size (mm)				0.000			0.426
Median	18.0 (9.0 ~ 35.0)	10.0 (7.0 ~ 17.0)	32.0 (21.0 ~ 42.0)		17.0 (9.1 ~ 28.0)	18.0 (10.0 ~ 30.0)	
≤ 10	470 (31.8%)	390 (52.2%)	80 (10.9%)		87 (27.6%)	80 (25.4%)	
11–30	531 (35.9%)	285 (38.2%)	246 (33.7%)		155 (49.2%)	161 (51.1%)	
31–50	372 (25.2%)	56 (7.5%)	316 (43.2%)		56 (17.8%)	58 (18.4%)	
> 50	105 (7.1%)	16 (2.1%)	89 (12.2%)		17 (5.4%)	16 (5.1%)	
No. of CTCs (FU/3 mL)	9.5 (6.6 ~ 11.3)	9.7 (7.1 ~ 11.5)	8.0 (6.2 ~ 11.2)	0.001	9.5 (6.8 ~ 11.2)	7.8 (6.1 ~ 11.0)	0.124
TgAb (IU/mL)	1.885 (1.053 ~ 11.863)	2.525 (1.120 ~ 25.623)	1.640 (1.030 ~ 5.670)	0.000	1.890 (1.110 ~ 16.010)	1.645 (1.030 ~ 7.073)	0.049
TPOAb (IU/mL)	1.445 (0.570 ~ 8.593)	1.515 (0.560 ~ 19.045)	1.425 (0.580 ~ 6.243)	0.463	1.520 (0.515 ~ 12.185)	1.490 (0.565 ~ 7.058)	0.818
Pathology				–			–
PTC	–	721 (96.5%)	–		296 (94.0%)	–	
Others	–	26 (3.5%)	–		19 (6.0%)	–	
*BRAF* V600E mutation*				–			
mutant	–	184 (73.0%)	–		79 (66.9%)		
wild	–	68 (27.0%)	–		39 (33.1%)		

*PSM* propensity score matching, *CTC* circulating tumor cell, *TgAb* thyroglobulin antibody, *TPOAb* Thyroid peroxidase antibody, *PTC* papillary thyroid carcinoma

^*^Before PSM, 252 cases detected. After PSM, 118 cases detected

Among the thyroid cancer patients included, 96.5% (721/747) of the tumor pathological type was papillary thyroid carcinoma (PTC) before PSM, and the remaining small portion was follicular thyroid carcinoma, anaplastic thyroid carcinoma and medullary thyroid carcinoma (shown in Table 1). Before PSM, the *BRAF* V600E mutation was detected in postoperative pathological specimens from 252 patients with thyroid cancer, of which 73.0% (184/252) were the mutant type and 27% (68/252) were the wild type (shown in Table 1). After PSM, PTC accounted for 94.0% (296/315) of the pathologic types in the malignant group. 118 patients with thyroid cancer were tested for the *BRAF* V600E mutation, of which 66.9% (79/118) were the mutant type and 33.1% (39/118) were the wild type (shown in Table 1).

### The diagnostic efficiency of CTC and other examination methods for thyroid cancer

**Fig. 2:**
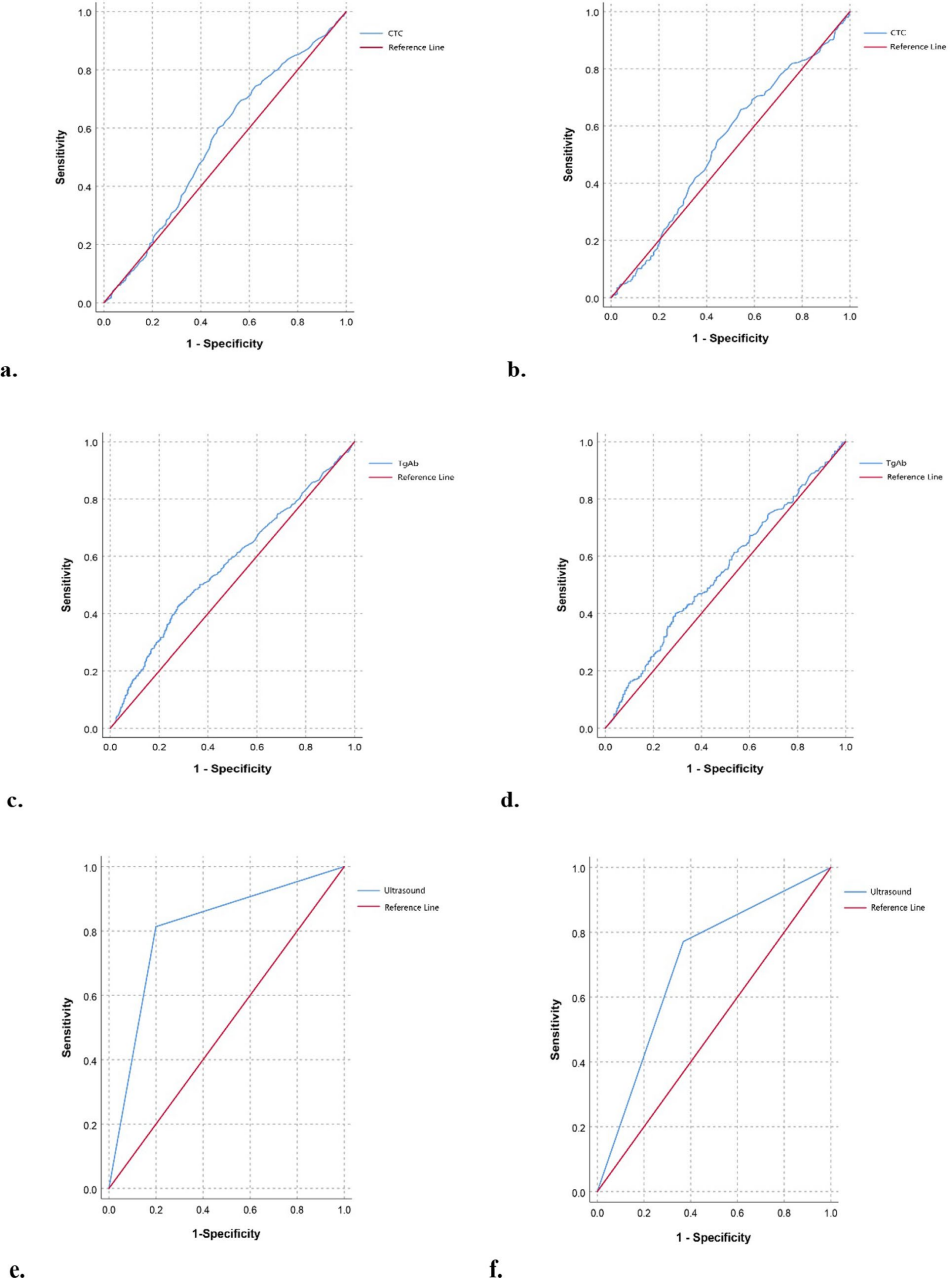

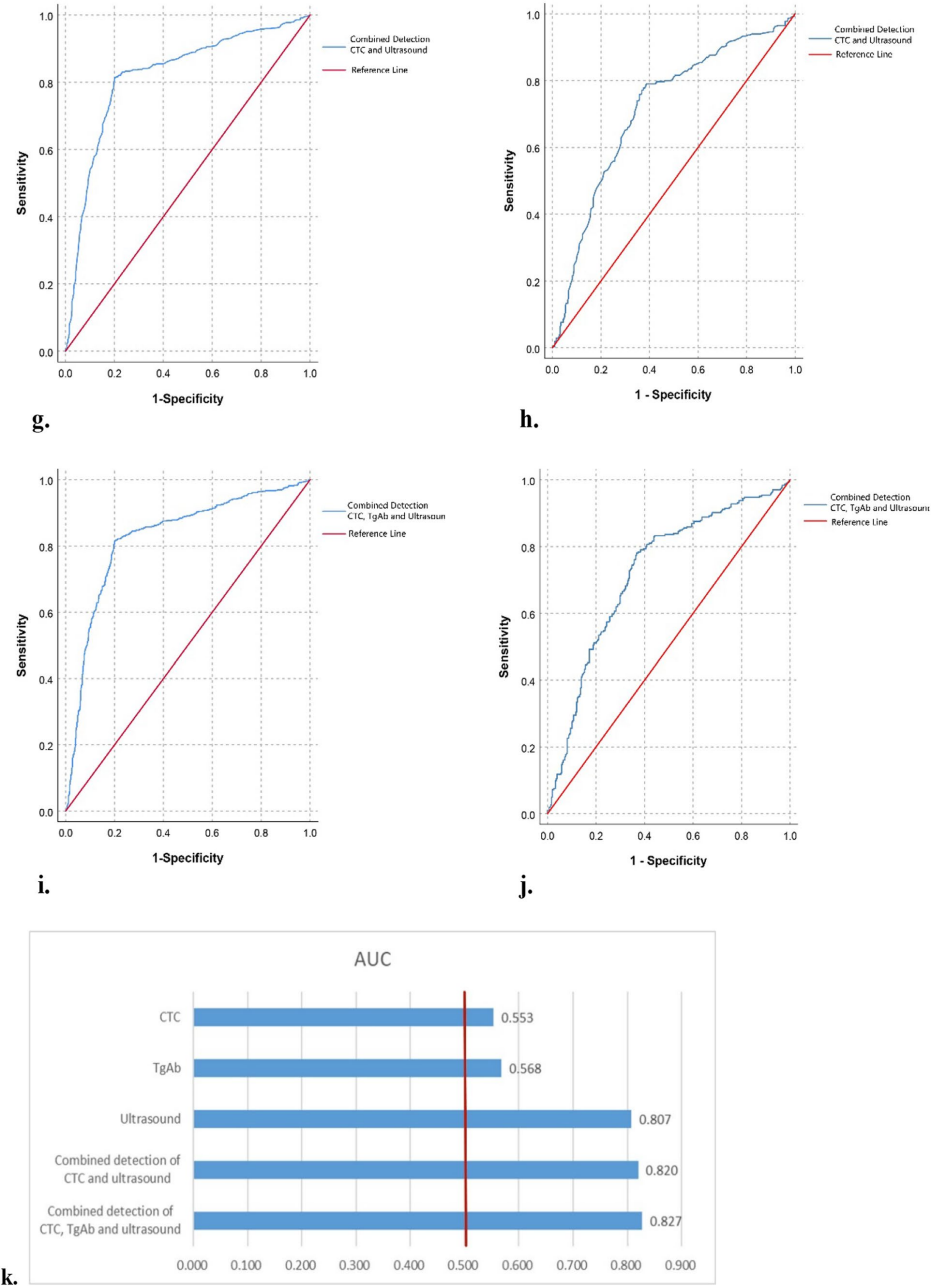

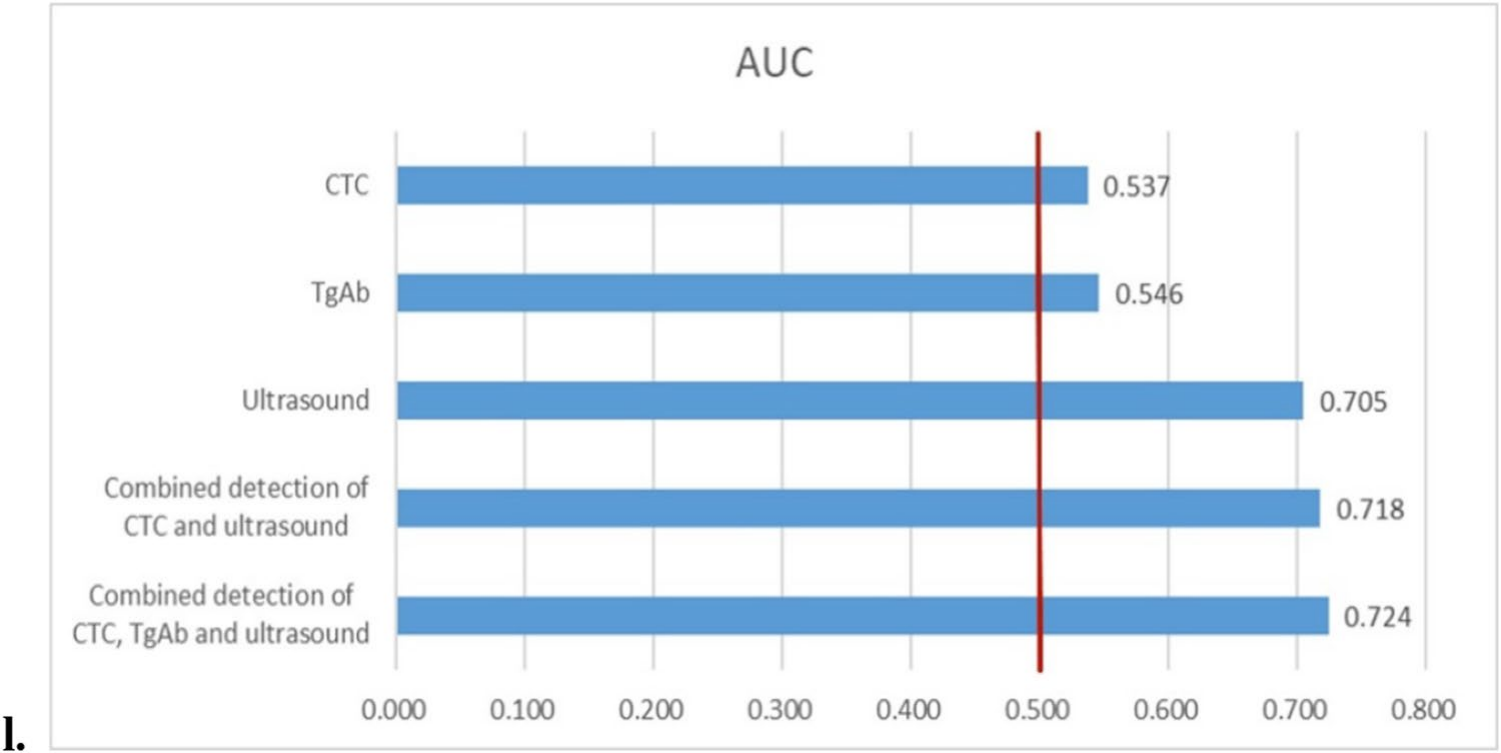
ROC curves of different indicators for predicting thyroid cancer before and after PSM. **a** ROC analysis of CTC before PSM. **b** ROC analysis of CTC after PSM. **c** ROC analysis of TgAb before PSM. **d** ROC analysis of TgAb after PSM. **e** ROC analysis of ultrasound before PSM. **f** ROC analysis of ultrasound after PSM. **g** ROC analysis of ‘CTC + ultrasound’ before PSM. **h** ROC analysis of ‘CTC + ultrasound’ after PSM. **i** ROC analysis of ‘CTC + TgAb + ultrasound’ before PSM. **j** ROC analysis of ‘CTC + TgAb + ultrasound’ after PSM. **k** AUC of different indicators before PSM. **l** AUC of different indicators after PSM. *ROC* receiver operating characteristic, *PSM* Propensity Score Matching, *CTC* circulating tumor cell, *TgAb* thyroglobulin antibody, *AUC* area under the curve

As shown in Fig. 2 and Table 2, the sensitivity and specificity of CTC, TgAb, color doppler ultrasound and their combinations were analyzed by ROC curve analysis. Before PSM, the areas under the curve (AUCs) of CTC, TgAb and ultrasound were 0.553, 0.568 and 0.807 (*p* < 0.001, compared to CTC), respectively. The AUCs of the combined detection of ‘CTC + ultrasound’ (combine 1) and combined detection of ‘CTC + TgAb + ultrasound’ (combine 2) were 0.820 (*p* = 0.040 compared to ultrasound alone) and 0.827 (*p* = 0.045 compared to combine 1), respectively. After PSM, the AUCs of CTC, TgAb and ultrasound were 0.537, 0.546 and 0.705 (*p* < 0.001, compared to CTC), respectively. The AUCs of combine 1 and combine 2 were 0.718 (*p* > 0.05 compared to ultrasound alone) and 0.724 (*p* > 0.05 compared to ultrasound alone, *p* > 0.05 compared to combine 1), respectively. In addition, FNA was performed in 120 patients with thyroid cancer and 30 patients with benign thyroid tumors in the population included in the present study. Before PSM, the sensitivity and specificity of FNA in the diagnosis of thyroid cancer were 78.3% and 100.0%, respectively. After PSM, 45 patients of the 315 patients included in the malignant group and 22 patients of the 315 patients included in the benign group were performed FNA. The sensitivity and specificity of FNA in the diagnosis of thyroid cancer were 77.8% and 100.0%, respectively.

**Table 2:** The diagnostic efficiency of different indicators for thyroid cancer before and after PSM

Variable	Before or after PSM	Cutoff	AUC	Sensitivity (%)	Specificity (%)
CTC (FU/3 mL)	B	7.55	0.553	69.5	43.5
	A	7.55	0.537	65.6	45.8
TgAb (IU/mL)	B	4.225	0.568	42.4	72.4
	A	3.580	0.546	40.0	70.8
Ultrasound	B	–	0.807	81.3	79.7
	A	–	0.705	77.1	63.2
CTC + ultrasound	B	–	0.820	81.4	80.0
	A	–	0.718	79.3	61.7
CTC + TgAb + ultrasound	B	–	0.827	81.5	80.0
	A	–	0.724	78.0	63.3

*PSM* Propensity Score Matching, *CTC* circulating tumor cell, *TgAb* thyroglobulin antibody, *AUC* area under the curve, *A* after PSM, *B* before PSM

### Relationship between CTCs and characteristics of thyroid carcinoma

**Fig. 3:**
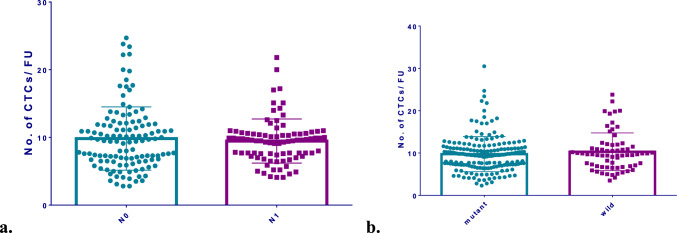
**a** No. of CTCs between the N0 and N1 subgroups. **b** No. of CTCs between the *BRAF* V600E mutant and wild subgroups. *CTC* circulating tumor cell

A total of 607 thyroid cancer patients of the 1478 patients included in this study underwent TNM system staging, including 338 in N0 stage (subgroup N0), 258 in N1 stage (subgroup N1), and 11 in Nx stage (shown in Table 3). There was no significant difference between subgroup N0 and subgroup N1 regarding the number of CTCs (shown in Fig. 3a). The *BRAF* V600E mutation was detected in the postoperative pathological specimens of 252 patients with thyroid cancer included in this study. Based on the presence or absence of *BRAF* V600E mutations, the patients were divided into mutant subgroup (*n* = 184) and wild subgroup (*n* = 68). Similarly, the number of CTCs was comparable in the two subgroups (shown in Fig. 3b).

**Table 3:** Baseline characteristics of 607 thyroid cancer patients undergoing TNM staging

Characteristics	All	N0	N1	Nx
*n*	607	338	258	11
Gender				
Male	118 (19.4%)	45 (13.3%)	70 (27.1%)	3 (27.3%)
Female	489 (80.6%)	293 (86.7%)	188 (72.9%)	8 (72.7%)
Age (years)	46 (35 – 53)	48 (40 – 54)	39 (31 – 50)	51 (36 – 63)
Tumor size (mm)	10.0 (7.0 – 17.0)	8.8 (6.0 – 13.0)	13.0 (9.0 – 22.8)	17.0 (8.0 – 27.0)
TgAb (IU/mL)	2.295 (1.120 – 20.893)	2.270 (1.120 – 17.670)	2.290 (1.100 – 34.320)	3.790 (1.298 – 16.000)
TPOAb (IU/mL)	1.520 (0.578 – 18.705)	1.820 (0.620 – 30.320)	1.220 (0.460 – 10.000)	1.910 (0.625 – 14.088)
Pathology				
PTC	598 (98.5%)	335 (99.1%)	254 (98.4%)	9 (81.8%)
Others	9 (1.5%)	3 (0.9%)	4 (1.6%)	2 (18.2%)

*CTC* circulating tumor cell, *TgAb* thyroglobulin antibody, *TPOAb* Thyroid peroxidase antibody, *PTC* papillary thyroid carcinoma

Factors with statistical differences before PSM in Table 1 were included in the multivariate regression analysis as independent variables. According to the results of binary logistic analysis, age and tumor size were independently associated with thyroid cancer (OR: 1.030, 95% *CI* 1.020–1.041; OR: 1.078, 95% *CI* 1.068–1.088, respectively) and lymphatic metastasis (OR: 0.953, 95% *CI* 0.938–0.968; OR: 1.058, 95% *CI* 1.039–1.077, respectively). However, the number of CTCs and TgAb were not the Independent influencing factors of thyroid cancer and lymphatic metastasis (shown in Tables 4 and 5).

**Table 4:** Binary logistic analysis of influence factors of thyroid cancer

Variable	*β*	*S.E*	*Wald*	*df*	*p* value	*OR* value	95% *CI*
Age (years)	0.030	0.005	35.452	1	0.000	1.030	[1.020–1.041]
Tumor size (mm)	0.075	0.005	256.059	1	0.000	1.078	[1.068–1.088]
No. of CTCs (FU/3 mL)	− 0.023	0.015	2.316	1	0.128	0.977	[0.948–1.007]
TgAb (IU/mL)	0.000	0.000	2.025	1	0.155	1.000	[0.999–1.000]

*CTC* circulating tumor cell, *TgAb* thyroglobulin antibody

**Table 5:** Binary logistic analysis of influence factors of lymphatic metastasis

Variable	*β*	*S.E*	*Wald*	*df*	*p* value	*OR* value	95% *CI*
Age (years)	− 0.048	0.008	37.155	1	0.000	0.953	[0.938–0.968]
Tumor size (mm)	0.056	0.009	36.982	1	0.000	1.058	[1.039–1.077]
No. of CTCs (FU/3 mL)	− 0.031	0.023	1.769	1	0.183	0.970	[0.926–1.015]
TgAb (IU/mL)	0.001	0.000	1.798	1	0.180	1.001	[1.000–1.001]

*CTC* circulating tumor cell, *TgAb* thyroglobulin antibody

## Discussion

As a part of liquid biopsy, CTC has been increasingly used in the preoperative diagnosis and postoperative follow-up of lung cancer, breast cancer, gastric cancer and other malignant tumors. FR + CTC technology can accurately and quantitatively detect CTCs in peripheral blood by identifying folate receptors in tumor cells and adopting targeted PCR technology, which provides a reliable reference for each diagnosis and treatment stage of tumor patients. To our knowledge, this is the largest sample size study to date to evaluate the diagnostic value of CTCs in thyroid cancer.

The results of this study showed that the incidence of both malignant and benign thyroid tumors was higher in females than in males, which was consistent with the epidemiological characteristics of thyroid cancer. The mechanism may be related to estrogen [10]. The age and maximum tumor diameter of the malignant group were smaller than those of the benign group before PSM. Because this was a retrospective study, all the cases included were preoperative patients. Benign tumors did not need surgery until they had symptoms of neck compression, so the maximum tumor diameter of the benign group was generally higher than that of the malignant group [11]. Moreover, benign tumors progressed slowly, and it often took many years for tumors to reach the size requiring surgery, so the age of the benign group was also higher than that of the malignant group. Despite this, the number of CTCs in the malignant group was still significantly higher than that in the benign group, which fully demonstrated that more tumor cells were released into the circulation even if the malignant tumor was small or existed in the body for a short time. Previous studies have confirmed that a certain amount of CTCs can be detected in the blood samples of patients with early- to mid-stage tumors [12], even earlier than imaging indications [13]. The results were consistent with previous studies. However, after balancing age and tumor size, the number of CTCs was comparable in the two groups. This result indirectly suggests that the patient's age and tumor size may affect the release of CTCs. A prospective study that included 72 patients with DTC and 30 healthy controls suggested that the mean number of CTCs in patients with DTC with distant metastases was significantly higher than that in the healthy controls [14]. Another study that enrolled 67 thyroid cancer patients showed that the number of CTCs in thyroid cancer patients was largely increased compared to that in normal controls [15]. There were also differences in age between the groups of the above studies. Different from the present study, the control groups of the above two studies were healthy controls rather than patients with benign thyroid tumors, and the CTCs detection methods were also different. It has been pointed out that compared with elderly patients, young patients with thyroid cancer were more likely to find tumor invasion and focal metastasis at the time of diagnosis [16]. This may explain why before PSM, the age and tumor diameter of the malignant group were smaller, but the CTCs value was higher. But this needs to be confirmed by further research.

At present, thyroid cancer is often diagnosed by improving thyroid function tests, related antibody detection, color doppler ultrasound examination, contrast-enhanced ultrasonography, and aspiration biopsy. As an emerging diagnostic method, most studies have reported the efficacy of CTCs in monitoring postoperative thyroid cancer recurrence and radioiodine therapy [5, 17, 18]. There are few reports on the preoperative diagnostic value of CTCs in thyroid cancer, and most published studies have focused on lung cancer. FR + CTC is one of the detection methods of CTC. According to Na’s study [19], the sensitivity and specificity of FR + CTC for cancer diagnosis in the elderly population were 85.7% and 65.0%, respectively, with 10.0 CTC FU/3 mL. In combination with serum tumor biomarkers, the diagnostic efficiency of FR + CTC was improved. Yu et al. reported that the sensitivity of FR + CTC was 73.2% for non-small cell lung cancer (NSCLC) diagnosis [20]. Nevertheless, a prospective, multicentre, cohort study including 614 participants in France demonstrated that the sensitivity of CTC detection for lung cancer detection was only 26.3%, and concluded that CTC was unable to predict lung cancer or extrapulmonary disease [21]. In our study, the results showed that with the cutoff value of 7.55 FU/3 mL, the sensitivity of FR + CTC for thyroid cancer diagnosis was 65.6% after PSM. Binary logistic analysis indicated that CTC was not an independent influencing factor in thyroid cancer. These results suggest that detecting CTCs alone has a certain diagnostic efficacy for thyroid cancer, but it is relatively weak. On the one hand, this may be related to the lower release of CTCs in the blood than other malignant tumors, owing to thyroid cancer being considered to be an inert tumor. On the other hand, it may also be relevant to the expression of FRs in thyroid cancer cells. Although several studies have confirmed the specific expression of FRs in malignant tumor cells [22–24], there is no report on the expression level of FRs in thyroid cancer cells compared with other tumors. Further analysis showed that after PSM, the sensitivities of ‘CTC + ultrasound’ and ‘CTC + TgAb + ultrasound’ were 79.3% and 78.0%, respectively, the AUCs of which were comparable with that of ultrasound alone. This means that the diagnostic efficiency of CTC combined with other routine examinations was not improved.

This study also analyzed the relationship between the number of CTCs and lymph node metastasis. There was no significant difference in the number of CTCs between the N0 and N1 subgroups. Additionally, the binary logistic analysis indicated that CTC was not an independent influencing factor in lymphatic metastasis. A recent study found that neither lymph node metastasis nor distant spread had impacts on the number of CTCs [15]. The reason for this result may be attributed to the fact that PTC mainly metastasizes through lymph nodes instead of blood. With the development of oncogene diagnosis, the *BRAF* V600E gene mutation is considered to be related to extracapsular invasion, lymph node metastasis, multifocality and therapeutic efficacy of thyroid cancer [25]. In the present study, we innovatively analyzed whether the *BRAF* V600E mutation affects the number of CTCs. The results suggested that the number of CTCs was not related to *the BRAF* V600E mutation.

The advantage of this study is that the sample size is relatively large, and PSM was use to balance the confounding factors. The disadvantage is that it is a retrospective, single-center study. Further prospective randomized controlled studies are needed to confirm this conclusion.

## Conclusion

In conclusion, though CTCs analysis is an objective, convenient, and noninvasive method compared to ultrasound, FNA and other common detection methods, its efficacy in diagnosing thyroid cancer is limited.

## Data Availability

The data that support the findings of this study are available from the corresponding author upon reasonable request.

## Abbreviations

cfDNA: Circulating free DNA
CTC: Circulating tumor cell
DTC: Differentiated thyroid carcinoma
FNA: Fine-needle aspiration
FR: Folate receptor
miRNA: MicroRNA
NSCLC: Non-small cell lung cancer
PCR: Polymerase chain reaction
PSM: Propensity Score Matching
PTC: Papillary thyroid carcinoma
TgAb: Tyroglobulin antibody
TPOAb: Thyroid peroxidase antibody
